# Non-rapid eye movement sleep with low muscle tone as a marker of rapid eye movement sleep regulation

**DOI:** 10.1186/1471-2202-7-2

**Published:** 2006-01-09

**Authors:** Gilberte Tinguely, Reto Huber, Alexander A Borbély, Peter Achermann

**Affiliations:** 1 Institute of Pharmacology and Toxicology, University of Zurich, Winterthurerstrasse 190, CH-8057 Zurich, Switzerland

## Abstract

**Background:**

It was recently reported that epochs of non-REM sleep (NREMS) with low muscle tone represent a partial correlate of REM sleep (REMS). To further investigate this phenomenon, episodes of restricted night-time sleep (23:00 – 03.00h) and subsequent morning sleep (10:00 – 13:00h) were analysed.

**Results:**

Epochs of NREMS with low muscle tone (NLMT) were identified. Their frequency was higher in morning sleep than in night sleep. At night, the latency to the first occurrence of NLMT showed a bimodal distribution with modes at sleep onset and close to REMS onset. In morning sleep, the distribution was unimodal with the mode at sleep onset. An episode of NLMT at sleep onset occurred in 35.5% of the night sleep episodes and in 60.9% of the morning sleep episodes without sleep onset REMS (SOREMS). Also SOREMS occurred predominantly in morning sleep. REMS episodes were longer and NREMS episodes shorter in morning sleep than in night sleep, whereas cycle duration did not differ. Simulating the time course of slow-wave activity revealed a close correspondence between empirical and computed values for night sleep, and some discrepancies for morning sleep.

**Conclusion:**

The results provide further evidence that NREMS with low muscle tone is a marker of REMS regulation. NLMT at sleep onset may represent an early manifestation of REMS.

## Background

REM sleep (REMS) is characterised by rapid eye movements, a mixed frequency, low amplitude EEG, and a low submental muscle tone with phasic twitches. At the transition from non-REM sleep (NREMS) to REMS these three features do not appear synchronously. The EMG level may decrease prior to the occurrence of the two other markers, and may also persist for some time after the end of REMS [1,2]. NREMS with low muscle tone (NLMT) was observed not only before and after REMS but also at sleep onset in a selective REMS deprivation study. NLMT was enhanced by total sleep deprivation and selective REMS deprivation [3]. It was proposed that epochs of NLMT could be correlates of REMS and therefore serve as markers of REMS regulation.

The aim of the present study was to further investigate the relationship between NLMT and REMS by analysing the first 3 h of a 4-h nocturnal sleep episode and a subsequent 3-h daytime sleep episode.

## Results

### REMS latency and SOREMS episodes

**Figure 1:**
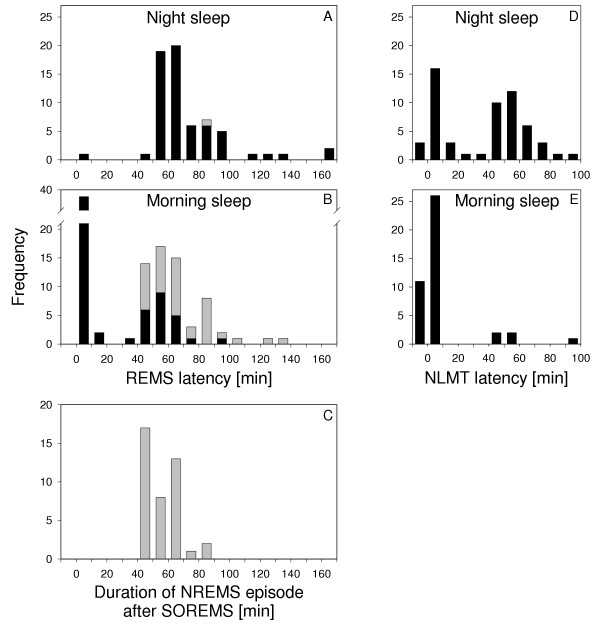
**Latency to REMS and NLMT**. Distribution of REMS latencies (black bars) in 63 night (**A**) and 63 morning (**B**) sleep episodes. Grey bars: latency to the second REMS episode (after a SOREMS episode) measured from sleep onset. **C**: Distribution of intervals (i.e. length of first NREMS episode) between SOREMS episodes and subsequent REMS episodes. Distribution of the latency to the first occurrence of low muscle tone in NREMS (NLMT) that occurred prior to the first REMS episode in 60 night (**D**) and 61 morning (**E**) sleep episodes. In 3 night and 19 morning sleep episodes, no NLMT was observed prior to the first REMS episode. Zero on the abscissa represents sleep onset defined as the first epoch of stage 2 or REMS. Negative values correspond to stage 1. Data were plotted in 10-min bins.

REMS latency showed a unimodal distribution with a maximum at 65 min during night sleep and a bimodal distribution with modes at 5 and 55 min and a trough at 25 min during morning sleep (Fig. 1A,B). One single sleep onset REMS (SOREMS; REMS latency < 20 min) episode occurred at night, while a total of 40 SOREMS episodes were observed during morning sleep (Table 1). At night, REMS latencies ranged from 0 to 164 minutes. The four longest latencies were from a single individual with a 'skipped' first REMS episode in all four nights (i. e. an episode with reduced SWA occurring at the expected time of REMS). In the morning, REMS latencies ranged from 0 to 99 minutes. The duration of NREMS episodes following a SOREMS episode ranged from 40 to 85 min (Fig. 1C); this range is comparable to that of REMS latencies at night (Fig. 1A).

Seven of 16 subjects showed SOREMS episodes in all four morning sleep episodes, one subject in three, three in two and three in one episode. Only two subjects had no SOREMS episode.

### Episodes of NLMT

**Figure 2:**
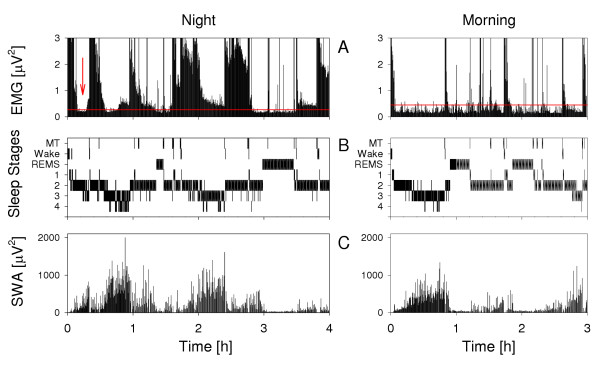
**Muscle tone, sleep stages and SWA**. Time course of (**A**) EMG variance (i.e. total power), (**B**) sleep profile (MT, movement time; 1 to 4, NREMS stages 1 to 4), and (**C**) slow-wave activity (SWA) of an individual for a night and a morning sleep episode. The horizontal red lines in A indicate the threshold that was predetermined to identify episodes of low muscle tone (see Methods). Note the episodes with low level of EMG variance during NREMS at sleep onset (red arrow) and later in the night. In the morning, the episodes of NLMT are longer than in night sleep and often, the muscle tone remained low during the entire recording.

Epochs of NREMS with low muscle tone (NLMT) are illustrated for one subject (Fig. 2). The latency to the first appearance of NLMT showed a bimodal distribution with modes at 5 and 55 min in night sleep (Fig. 1D). The first mode was centred at sleep onset and the second mode was situated in the proximity of REMS onset. The distribution closely resembled the distribution of REMS latencies in morning sleep. A unimodal distribution with a mode at 5 min was observed in morning sleep (Fig. 1E). Epochs of NLMT were more frequent in morning sleep than in the first 3 hours of night sleep (Table 1; note that in Fig 1E the sleep episodes with SOREMS are also included).

In night sleep, 22 episodes of NLMT at sleep onset were observed (Table 1). The one subject with four 'skipped' first REMS episodes at night showed neither SOREMS episodes nor episodes of NLMT at sleep onset. The other subject without SOREMS episodes had no episode of NLMT at sleep onset at night but three in the morning.

In 14 of the 23 morning sleep episodes without SOREMS, episodes of NLMT at sleep onset were observed. Their length was similar in night and morning sleep.

### Slow wave activity

**Figure 3:**
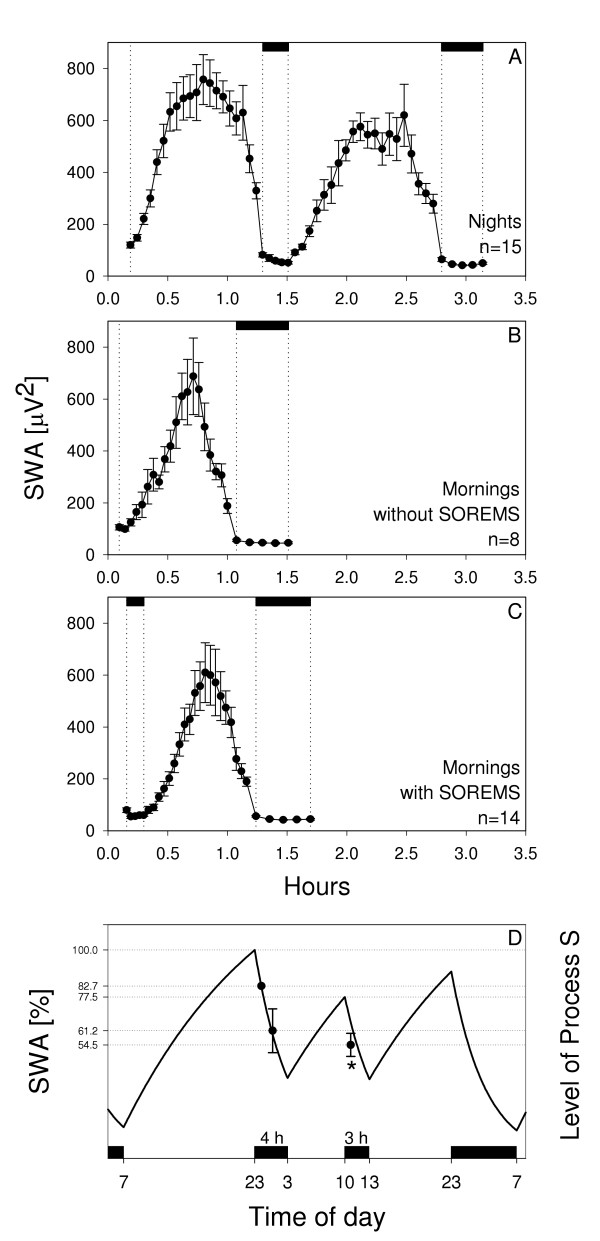
**SWA and Process S**. Time course of slow-wave activity (SWA) in night (**A**) and morning sleep episodes (**B**: mornings without SOREMS episodes; **C: **mornings with SOREMS episodes). NREMS episodes were subdivided into 20 equal intervals and REMS episodes into 5 equal intervals. Mean values per interval were calculated within subjects prior to averaging across subjects (mean ± SEM). For night sleep, only data of recordings with 2 complete cycles were included. Black bars at the top delineate REMS episodes. **D**: Simulation of homeostatic Process S (solid line) and time course of empirical slow-wave activity (SWA) in consecutive NREMS episodes (filled circles; ± 95 % confidence interval). The level of S at sleep onset of the 4-hour night-time episode was set to 100 %. Based on the average timing of the first NREMS episode and the previously defined time constants [4], the level of Process S at episode midpoint of the first NREMS episode was calculated as 82.7 %. This value equals average SWA of first NREMS episode and served as the reference value for the calculation of relative SWA in the second NREMS episode of the night (61.2 %) and the first NREMS episode of the morning (54.5 %). * S outside the 95% confidence interval of SWA data. Black bars denote sleep episodes.

The mean time course of SWA during night and morning sleep is plotted in Fig. 3. Mean SWA in the first NREMS episode of morning sleep was lower than in the first NREMS episode of night sleep (Table 1). Slow-wave energy (cumulative SWA in NREMS) of the first NREMS episode was also lower in the morning than at night.

Simulations of Process S were performed (Fig. 3D) to test whether the changes in SWA were in accordance with the two-process model of sleep regulation [4]. Process S represents the homeostatic (i.e., sleep-waking dependent) process of sleep regulation. Its time course was derived from EEG SWA [4]. The simulations were based on a regular sleep-wake schedule (sleep: 23:00 to 7:00) followed by a 4 h sleep episode at night (23:00 to 3:00) and a 3 h daytime sleep episode (10:00 to 13:00). The time constants (increase of S: 18.2 h; decrease of S: 4.2 h) corresponded to those used in Daan et al. [4]. The simulations revealed a reduced level of S (sleep propensity) at sleep onset in the morning (77.5 %) compared with the level at sleep onset at night (100 %). For night sleep, a close correspondence between empirical SWA and the simulated level of S was observed (i.e., S was within the 95 % confidence interval of empirical SWA of the second NREMS episode). In the first NREMS episode of morning sleep, however, the predicted level of S was above the empirical values (S outside 95 % confidence interval).

### Sleep variables derived from visual scoring (Table 2)

Waking after sleep onset, the duration and percentage (% of total sleep time) of REMS and stage 1 were higher in the morning than at night, whereas the amount of stages 2, 3 and 4 was lower. Sleep latency was shorter in the morning than at night, whereas REMS latency did not differ if SOREMS was excluded. Total sleep time, sleep efficiency, and movement time did not differ between night and morning sleep episodes.

### Cycle and episode duration (Table 2)

Sleep cycle duration did not differ between night and morning sleep. REMS episodes, however, were longer and NREMS episodes shorter in morning sleep than in night sleep.

## Discussion

### Episodes of NLMT

The present study aimed at clarifying the relationship between a particular feature of NREMS, NLMT, and REMS regulation by analysing restricted nocturnal sleep and subsequent daytime sleep. The results provide evidence that NLMT is determined by REMS propensity.

NLMT occurred more frequently in morning sleep than in night sleep. In morning sleep, the latency to the first epoch of NLMT showed a unimodal distribution. In night sleep, a bimodal pattern prevailed with modes at sleep onset and in the proximity of REMS onset (Fig. 1D). This distribution corresponds to that of REMS latency in morning sleep (Fig. 1B). It is also similar to the pattern that had been observed in a selective REMS deprivation protocol [3]. The duration of the episodes of NLMT at sleep onset was slightly longer than the duration of SOREMS episodes and their latency was shorter. Low muscle tone usually precedes and outlasts REMS [1-3]. From these results, we conclude that NLMT may represent a window for SOREMS. If the episodes of NLMT at sleep onset would just represent the normal wake-sleep transition then muscle tone would be expected to gradually decrease [2,5]. As illustrated in Figure 2 (left) both decrease and increase of muscle activity at sleep onset were typically rather sudden. No specific EEG markers were found to accompany episodes of NLMT as revealed by gross visual inspection.

When NREMS and REMS were considered together, 86% of the morning sleep episodes, but only 37% of the night sleep episodes started with low muscle tone. In morning sleep both the circadian drive [6-8] and the reduced NREMS pressure contributed to a high REMS propensity. Low muscle tone may be considered a REMS marker irrespective of the manifestation of this sleep state and epochs of low muscle tone at sleep onset may reflect the early appearance of REMS. If the drive for REMS is low, NLMT may be the only manifestation of this sleep state. NLMT may therefore be regarded as a facet of REMS regulation as previously proposed [3]. In that study, selective REMS deprivation increased NLMT in recovery sleep and NLMT was higher in daytime sleep than in the baseline night [3]. In our study, the number of episodes of NLMT increased, in parallel to SOREMS and REMS, in morning sleep compared to night sleep. The present findings challenge the concept of discrete states and favours the concept of interleaved states [3,9,10]. Our hypothesis could, for instance, further be tested in a forced desynchrony protocol that allows to separate homeostatic and circadian components [8].

Low muscle tone is required to score REMS [11]. When speaking of muscle tone or EMG, sleep researchers usually refer to recordings of submental or mental muscle activity, which has a state-specific tone [12]. One has to keep in mind that the muscle tone of trunk and limb muscles shows a different pattern than the muscle tone of head and neck muscles: the reduced tonic level during sleep remains rather stable throughout the night without further decrease in REMS [13]. Cells in the medial brainstem reticular formation are thought to control motor movement [14]. They are active during waking and REMS while during NREMS their activity is reduced [14]. During REMS motoneurons in the brainstem are tonically inhibited although central motor systems are highly active [14]. Motoneuron hyperpolarisation and ensuing loss of muscle tone are due to a combination of disfacilitation and inhibition by the co-ordinated action of GABA and glycine release onto the motoneurons and concomitant decrease of norepinephrine and serotonin release onto them [14]. If our assumption is correct, then the activity of the motoneurons during episodes of NLMT should show a similar pattern as seen during REMS.

### SOREMS

The high number of SOREMS episodes in morning sleep (Fig. 1B) showed that this phenomenon is common in experimental protocols and illustrates that there may indeed be a REMS window at sleep onset. SOREMS episodes and increased amounts of REMS were reported for subjects sleeping in the morning after a night with or without sleep [15,16]. In a time-free environment [7] and in a forced desynchrony protocol [8], SOREMS occurred at a circadian phase corresponding to morning sleep. Their number decreased in naps scheduled throughout daytime hours (from morning to evening) [17]. After spontaneous wakefulness in a long scotoperiod protocol, SOREMS episodes were more frequent in the early morning hours [18]. Not only circadian factors but also NREMS pressure influences SOREMS propensity. Thus sleep initiated at 7:00 h after total sleep deprivation did not result in increased REMS [19,20]. In the present study, SOREMS and REMS were enhanced in morning sleep by the high circadian drive and the reduced NREMS propensity. The preceding restricted nocturnal sleep episode not only reduced NREMS propensity (Fig. 3D) but also induced a partial REMS deprivation, as a large portion of REMS occurs in the second half of the night. The shorter sleep latency in the morning than at night may be due to the increased REMS propensity.

### Slow wave activity

Simulations with the two-process model of sleep regulation [4] revealed a close correspondence between empirical SWA and the level of S for night sleep. However, the empirical values of SWA were below the predicted level of S in the first NREMS episode of morning sleep. This discrepancy between the data and the model is in accordance with findings of Beersma and co-workers [21] who reported reduced intensity of NREMS under conditions of increased REMS pressure. As already discussed, the 4-h sleep episode in the previous night decreased NREMS pressure, which did not increase again up to the level of night sleep because the time between the night and the morning sleep episode was too short. Concomitantly, the sleep restriction and the circadian phase increased REMS pressure. Thus, increased REMS propensity appears to inhibit the full manifestation of SWA in morning sleep. Therefore not only a high NREMS pressure impedes REMS, but also high REMS drive lowers NREMS intensity [22]. Thus, SWA represented in the model by Process S is not just dependent on sleep-wake history, but additionally on the balance between NREMS and REMS pressure.

## Conclusion

We conclude that NLMT is a marker of homeostatic and circadian REMS regulation. At sleep onset, the epochs of low muscle tone represent an early manifestation of REMS. Reduced NREMS pressure associated with enhanced homeostatic and circadian REMS drive in the morning can account for the higher frequency of SOREMS episodes and epochs of NLMT in morning sleep than in night sleep.

## Methods

Data of 16 healthy young right-handed men (mean age 22.3 years, range 20 – 25 years) who slept in the laboratory four times (sessions) at weekly intervals for 4 hours at night (beginning at either 22:45 or 23:15) and 3 hours in the subsequent morning (beginning at either 9:45 or 10:15) were analysed. Between the two sleep episodes the subjects remained in the laboratory and were under constant supervision. Subjects were requested to maintain the habitual sleep-wake schedule (23:00 – 07:00) on the night preceding the experimental night. Compliance was verified by means of wrist-worn activity monitors. The subjects gave informed written consent, and the study protocol was approved by the Medical-Ethical Committee "Physiology-Pharmacology" of the University of Zurich. Three of the four sessions are from an experiment investigating the effect of electromagnetic fields (EMF) of mobile phones on sleep and the sleep EEG [23]. In the fourth week, the right hand of the subjects was vibrated intermittently (20 min on; 10 min off) during three hours prior to the morning sleep episode (unpublished data); ten minutes after the end of vibration, lights were switched off. All manipulations were performed only before the morning sessions.

We first tested whether experimental treatment (sessions) affected the variables of interest. Morning sleep episodes were subjected to one-way ANOVAs for repeated measures with the factor 'session' (1 to 4). All variables analysed except sleep latency (mean ± SEM of the four sessions: 5.7 ± 0.9, 5.3 ± 1.1, 3.0 ± 0.6, 6.9 ± 0.8 min) did not differ between the sessions. Furthermore, the distribution of the values of the variables in the four sessions did not give any indication of an effect of treatment. Therefore, we pooled the data of the four sessions and focused our analysis on the comparison of night and morning sleep episodes. Mean values were calculated within subjects prior to averaging across subjects. Data of the first 3 hours of night sleep were compared with the 3-hour morning sleep episodes using two-tailed paired t-tests.

Polysomnographic recordings were performed during all sleep episodes. EEG, submental EMG and EOG signals were conditioned by the following analog filters: a high-pass filter (-3 dB at 0.16 Hz), a low-pass filter (-3 dB at 102 Hz, <-40 dB at 256 Hz), and a notch filter (50 Hz). Data were sampled with a frequency of 512 Hz, digitally filtered (EEG and EOG: low-pass FIR filter, -3 dB at 49 Hz; EMG: band-pass FIR filter, -3 dB points at 15.6 and 54 Hz), and stored with a resolution of 128 Hz. Sleep stages were visually scored according to standard criteria [11]. Due to technical problems one night recording of a subject and one morning recording of another subject were incomplete and excluded from data analysis. The sleep cycles, NREMS and REMS episodes were defined according to the criteria of Feinberg and Floyd [24]. REMS episodes with latency shorter than 20 min were defined as sleep onset REMS (SOREMS) episodes.

EMG variance (i.e. total power) was calculated for consecutive 20-s epochs after elimination of ECG artefacts, i. e. excluding 30 data points before and after the occurrence of an R-wave. Epochs of low muscle tone were identified when the variance of the EMG was below the 90^th ^percentile level of the EMG variance in REMS epochs (Fig. 2). The threshold was determined separately for each sleep episode. All thresholds were visually controlled. In one recording, the signal-to-noise ratio of the EMG changed in the course of the sleep episode and the threshold had to be adjusted. Fifty-eight night-morning pairs were compared; 6 pairs had to be excluded because in one or both recordings the signal-to-noise ratio deteriorated and prevented a quantitative analysis. From the latter, 2 morning sleep recordings could be analysed automatically and additional two night and one morning sleep episodes by visual inspection. These data were only used for the analysis of NLMT at sleep onset. Episodes of NLMT with latency not longer than 20 min are referred to as NLMT at sleep onset. NLMT latency was defined as the interval between sleep onset (first epoch of stage 2) and the first appearance of NLMT.

Power spectra of consecutive 20-s epochs (FFT routine, Hanning window, averages of five 4-s epochs) were computed for derivation C3A2. Artefacts were identified by visual inspection and a semi-automatic procedure (see [23] for details). Analysis was restricted to slow-wave activity (SWA; EEG power in the 0.75 – 4.5 Hz range).

## List of abbreviations

REMS: rapid eye movement sleep

NREMS: non rapid eye movement sleep

SOREMS: sleep onset REMS (REMS latency < 20 min)

NLMT: NREMS with low muscle tone

EMG: electromyogram

EEG: electroencephalogram

ECG: electrocardiogram

SWA: slow-wave activity (EEG power in the 0.75 – 4.5 Hz range)

SWE: cumulative SWA in NREMS

FIR: finite impulse response

## Authors' contributions

GT analysed the data; RH, AAB and PA designed and executed the original experiment; GT, AAB and PA interpreted the data; all authors contributed to the draft and approved the manuscript.

## Figures and Tables

**Table 1:** SOREMS episodes, episodes of NLMT, slow wave activity

	Night sleep	Morning sleep
**SOREMS episodes**		
Number/total number of sleep episodes	1/63	40/63
Duration [min], mean ± SEM, n = 14		12.8 ± 2.8
Latency [min], mean ± SEM, n = 14		7.6 ± 1.3
**Frequency of epochs of NLMT **(n = 16)		
Number/number of epochs of NREMS [%]	19.7 ± 3.1	36.2 ± 5.0*
**Episodes of NLMT at sleep onset**		
Number/total number of sleep episodes without SOREMS	22/62	14/23
Duration [min], mean ± SEM	19.2+6.6 (n = 12)	19.9 ± 6.3 (n = 7)
Latency [min], mean ± SEM	6.4 ± 1.2 (n = 12)	1.5 ± 0.5 (n = 7)
**Slow-wave activity **(n = 16)		
SWA [μV^2^] mean ± SEM	552.6 ± 42.3	361.3 ± 33.7**
SWE [μV^2^s] mean ± SEM	39772.3 ± 4220.0	20293.7 ± 1853.0**

SOREMS: Sleep Onset REMS episodes (REMS episodes with latency < 20 min). NLMT: NREMS with low muscle tone. For NLMT at sleep onset, only sleep episodes without SOREMS were considered. SWA: mean slow wave activity (EEG power in the 0.75 – 4.5 Hz range) in the first NREMS episode. SWE: slow wave energy (cumulative SWA in NREMS) in the first NREMS episode.

* p < 0.01, ** p < 0.001; comparison of night and morning sleep episodes by paired t-tests

**Table 2:** Sleep variables derived from visual scoring

Variable	Night [min]	Night [%]	Morning [min]	Morning [%]
Total sleep time	162.92 ± 1.76		165.76 ± 0.97	
Sleep efficiency		90.51 ± 0.98		92.37 ± 0.53
WASO	2.39 ± 0.61		5.30 ± 0.83**	
Sleep latency	11.47 ± 1.38		5.28 ± 0.58**	
REMS latency	72.26 ± 5.54		58.18 ± 4.00°	
Movement time	3.28 ± 0.36		2.94 ± 0.30	
Stage 1	7.22 ± 0.91	4.56 ± 0.62	12.99 ± 1.80*	7.93 ± 1.10*
Stage 2	93.01 ± 4.29	57.34 ± 2.90	85.50 ± 4.97*	51.55 ± 3.00*
Stage 3	29.43 ± 2.79	17.88 ± 1.60	16.29 ± 2.41**	9.86 ± 1.45**
Stage 4	9.55 ± 2.97	5.76 ± 1.78	3.77 ± 1.57*	2.30 ± 0.95*
SWS	38.98 ± 4.37	23.64 ± 2.52	20.06 ± 3.53**	12.16 ± 2.14**
NREMS	131.99 ± 2.51	80.99 ± 1.22	105.56 ± 3.73**	63.71 ± 2.25**
REMS	23.71 ± 2.17	14.46 ± 1.28	47.21 ± 2.70**	28.36 ± 1.61**

Sleep cycle	89.7 ± 2.4		88.4 ± 4.1	
NREMS episode	70.0 ± 2.1		55.62.0**	
REMS episode	19.7 ± 1.5		32.7 ± 3.7*	

Mean values and standard errors (SEM) are reported (n = 16, 63 night and morning recordings of 16 subjects, except for sleep cycle duration: n = 15, one subject was excluded because of a 'skipped' first REMS episodes in all 4 nights). Mean values were first calculated within subjects prior to averaging across subjects. Stages are in min and in % of total sleep time. For night sleep, only the three first hours after lights out were used for the analyses. Statistics: comparison of night and morning sleep episodes by paired t-tests, * p < 0.01, ** p < 0.001

°n = 9, only subjects with at least one morning sleep episode without SOREMS (Sleep Onset REMS episodes: REMS episodes with latency < 20 min) were included.

WASO: waking after sleep onset; SWS: stages 3 and 4, NREMS: stages 2,3 and 4; REMS latency: time from sleep onset (first occurrence of stage 2) to the first occurrence of REMS.
